# Persistent Lactate Elevation in a Patient with Asthma Exacerbation and a Congenital Portosystemic Shunt: A Case Report and Literature Review

**DOI:** 10.3390/reports8010008

**Published:** 2025-01-17

**Authors:** Wing Fai Li, Bailey Fink, Rehnuma Khan, Xinmiao Luo, Muhammad Fahimuddin

**Affiliations:** 1 Department of Internal Medicine, Jacobi Medical Center, Bronx, NY 10461, USA; liw4@nychhc.org (W.F.L.);; 2 Albert Einstein School of Medicine, 1300 Morris Park Ave, Bronx, NY 10461, USA; bailey.fink@einsteinmed.edu (B.F.); rehnuma.khan@einsteinmed.edu (R.K.)

**Keywords:** type B lactic acidosis, congenital portosystemic shunt, asthma, beta-adrenergic agonist

## Abstract

**Background and Clinical Significance**: When lactate production surpasses the body’s clearance capacity, hyperlactatemia (lactate ≥ 2 mmol/L) or lactic acidosis (lactate ≥ 4 mmol/L) can develop. Lactic acidosis is classified into type A, which arises from regional or global tissue hypoperfusion, and type B, resulting from metabolic disturbances without tissue hypoxia. Type A lactic acidosis, often associated with conditions like sepsis or shock, is a critical marker of life-threatening conditions, whereas type B lactic acidosis is less frequently recognized in clinical practice. **Case Presentation**: A 95-year-old man presents with an asthma exacerbation and is treated with an albuterol inhaler. However, he is found to have persistently high lactate levels. Further investigation reveals a congenital intrahepatic portosystemic shunt on imaging. This, in conjunction with the ongoing use of beta-adrenergic receptor agonists, contributes to the development of type B lactic acidosis. **Conclusions**: The impact of lactic acidosis depends on its severity and clinical context. While beta agonists are a recognized cause of type B lactic acidosis, a potential role for structural liver abnormalities in reduced lactate clearance must be examined further.

## 1. Introduction and Clinical Significance

Lactic acid is widely used to assess clinical status and as a prognostic indicator. Mortality is nearly three times higher in patients with lactic acidosis in the context of low-flow states or sepsis, with prognosis worsening as lactate levels rise [1]. Physiologically, lactic acid is produced in various tissues, including the skin, red blood cells, brain, muscle, and gastrointestinal tract, with skeletal muscles generating the most during intense exercise [2]. An estimated 20 mmol/kg of lactate is produced in the human body daily, primarily metabolized through the Cori cycle for gluconeogenesis in the liver and kidneys [2]. Following glycolysis, pyruvate can either enter the citric acid cycle as acetyl coenzyme A (acetyl-CoA) or, in low-oxygen conditions, be converted to lactate [3]. When lactate production surpasses the liver or kidneys’ capacity to process it, lactate levels rise [4]. Hyperlactatemia is defined as lactate above 2 mmol/L, while levels above 4 mmol/L represent lactic acidosis. The impact of lactic acidosis depends on its severity and clinical context: elevated lactate may indicate impaired oxygen delivery or tissue oxygen utilization (type A lactic acidosis) or arise from other metabolic disturbances unrelated to oxygenation (type B lactic acidosis) [5]. While it is essential to rule out life-threatening type A lactic acidosis such as sepsis in every patient presenting with elevated lactate, the significance of type B lactic acidosis should not be underestimated.

Herein, to the best of our knowledge, we present a case of type B lactic acidosis in a 95-year-old with concurrent beta-adrenergic use and a congenital intrahepatic portosystemic shunt. By reviewing different etiologies and underlying the pathophysiology of different type B lactic acidosis and summarizing published cases of type B lactic acidosis, we aim to enhance clinical understanding of the causes and presentations of type B lactic acidosis.

## 2. Case Presentation

A 95-year-old Hispanic male presented to the emergency department with an acute onset of sputum production and a cough associated with occasional chest pain. His past medical history included hypertension and mild persistent asthma, well-controlled with a home-medication regimen of a budesonide/formoterol inhaler (one puff daily). He rarely used his albuterol inhaler as a rescue medication, with his last asthma exacerbation occurring nine months prior. He reported being in his usual state of health until 3–4 days before presentation when he experienced an increased productive cough, episodic wheezing, and mild dyspnea. He had been using his albuterol inhaler with some relief. He denied having fever, chills, or increased nighttime awakenings. He had recently attended a seniors’ camp where some individuals exhibited upper respiratory symptoms. The patient was otherwise independent in daily activities, did not use ambulatory aids, and had no history of diabetes, seizures, or strokes. He denied smoking, alcohol, or recreational drug use.

On examination in the emergency department, he was alert, oriented, and in no acute distress. His blood pressure was 136/61 mmHg, heart rate was 99 beats per minute, and oxygen saturation was 95% on room air. Physical examination revealed bilateral wheezing without accessory muscle use and no crackles or rales. The initial workup was negative for COVID-19, influenza A/B, respiratory syncytial virus, and adenovirus. The venous blood gas revealed a pH of 7.33 (normal 7.32–7.43), bicarbonate of 22 mmol/L (normal 22–29), and an anion gap of 10.1 mEq/L (normal ≤ 13.9). Serum lactate was 4.2 mmol/L (normal 0.3–1.3), with baseline lactate 1.6–2.4 mmol/L (normal 0.3–1.3). Troponin was mildly elevated at 31 ng/L, with no leukocytosis (white blood cell count of 5.34/nL) and normal procalcitonin (0.07 ng/mL). A chest X-ray showed no acute consolidation or infiltrates, and an electrocardiogram revealed normal sinus rhythm with a known right bundle branch block (RBBB). Peak expiratory flow (PEF) was measured at 180 mL. The patient received an albuterol nebulizer, ipratropium bromide nebulizer, intravenous magnesium, and 10 mg of intravenous dexamethasone for asthma exacerbation. An ipratropium bromide nebulizer was added as it was shown to be associated with fewer hospitalizations and improved PEF [6]. He was subsequently admitted to the medical floor for further management.

On the medical floor, the patient was maintained on an ipratropium bromide/albuterol inhaler every four hours and initially received oral prednisone of 40 mg, later transitioned to 40 mg of intravenous methylprednisolone every eight hours for two doses. Due to his significant lactic acidosis, a 500 mL bolus of lactated Ringer’s solution was administered. His vital signs and symptoms were closely monitored for any signs of sepsis. Throughout his hospital stay, the patient maintained satisfactory oxygen saturation on room air without requiring supplemental oxygen. He reported no fever, chest pain, shivering, or unusual movements, and lab results showed stable leukocyte and hemoglobin levels. Repeat serum lactate levels remained elevated at 4.3 mmol/L despite fluid administration, while troponin levels trended down from 31 ng/L to 26 ng/L. Despite the continued lactate elevation, the patient demonstrated clinical improvement, with low suspicion of end-organ dysfunction or sepsis.

**Figure 1:**
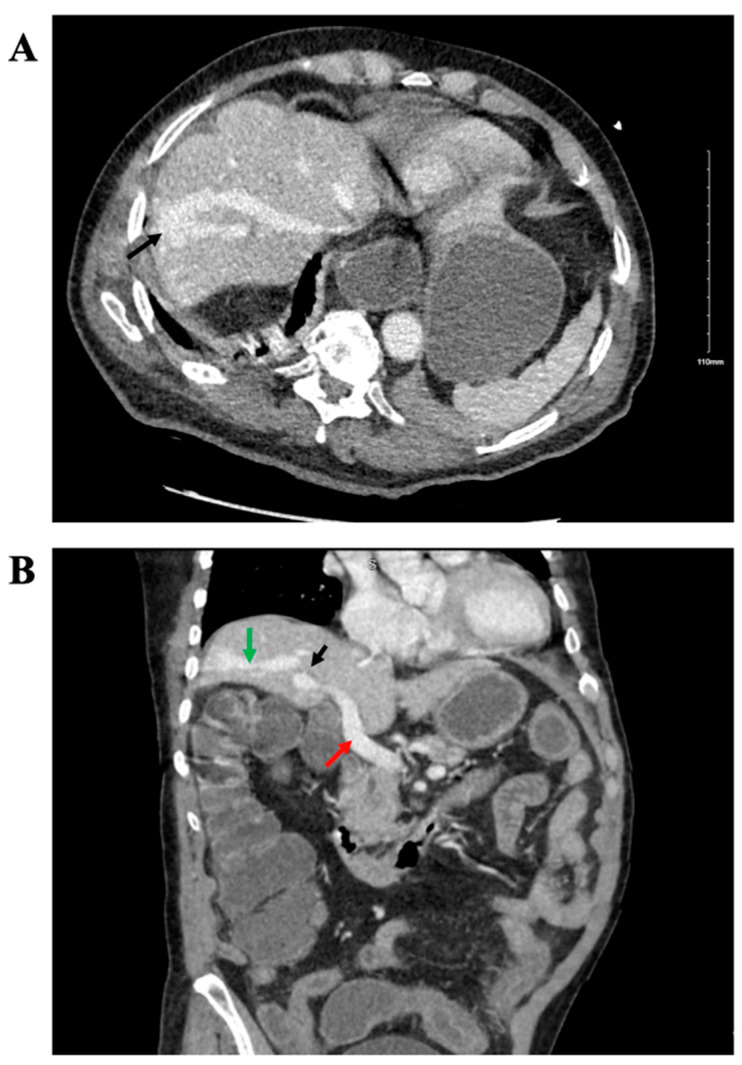
Computed tomography (CT) imaging with contrast demonstrated a portosystemic shunt. (**A**) Axial view of CT abdomen/pelvis highlighting a stellar dilated vascular lesion at the right hepatic lobe (black arrow). (**B**) Sagittal view of CT abdomen/pelvis showing communication between portal vein (red arrow) and hepatic vein (green arrow). The location of the shunt is indicated by the black arrow.

Type B lactic acidosis was suspected due to the clinical context. Serum thiamine levels were measured and found to be normal (111.3 nmol/L). An extensive review of the patient’s medical history revealed no evidence of malignancy, diabetes, AIDS, seizures, or past surgeries. His home medications included aspirin 81 mg daily, an albuterol inhaler, budesonide/formoterol inhaler, tamsulosin of 0.4 mg daily, lisinopril of 20 mg daily, and nifedipine of 30 mg daily. Apart from beta-adrenergic agonist use, there was no use of drugs known to cause lactic acidosis, such as metformin, cyanide, salicylates, linezolid, propofol, dideoxide, isoniazid, or toxins like ethylene glycol, methanol, and propylene glycol. His only previous admission was nine months earlier for pneumonia, COVID-19, and rotavirus infection, during which he was treated with piperacillin-tazobactam, dexamethasone, remdesivir, and an albuterol/ipratropium bromide inhaler. At that time, his serum lactate was 3.1 mmol/L initially, rising to 3.7 mmol/L, but it remained elevated at 2.4 mmol/L after the resolution of sepsis. Notably, a computed tomography (CT) of the abdomen and pelvis was done at that time and revealed an aberrant vessel connecting the portal vein and hepatic vein in the right liver lobe (Figure 1), suggesting a congenital intrahepatic portosystemic shunt, of which the patient was previously unaware. He confirmed again that he had no abdominal surgery or trauma in the past.

During his hospital stay, the patient showed no signs of jaundice, hepatic encephalopathy, cirrhosis, abnormal behavior, or seizures, which could indicate increased ammonia shunting to the brain. Serologic and biochemical tests showed no microcytic anemia, and normal blood urea nitrogen, albumin, and glucose levels. His liver function tests were normal. Ultimately, the patient’s asthma exacerbation was resolved, and he was discharged. A follow-up phone call conducted four months later revealed that the patient had returned to his baseline health. He had remained active and traveled back to his home country for vacation. He reported using his budesonide/formoterol inhaler one puff daily without experiencing any shortness of breath. No new laboratory tests or imaging studies were performed, and he was advised to follow up with his primary care provider in two months.

### Discussion

Since the case series of lactic acidosis was published in 1961, serum lactate levels have become an essential clinical marker for assessing tissue perfusion and monitoring shock [6]. Lactic acid exists in two forms: L-lactic acid and D-lactic acid [7]. D-lactic acid, produced by gut microbiota, is typically normal but can be elevated in conditions such as short bowel syndrome and jejunoileal bypass surgery [8]. In contrast, L-lactic acid, an endogenous metabolite, has greater clinical significance due to its strong correlation with tissue perfusion and mortality [7].

Lactate levels in the body are regulated by a balance between production and clearance. When production outpaces clearance or metabolism is impaired, lactic acid accumulation occurs. Type A lactic acidosis results from inadequate oxygen delivery to tissues, commonly due to shock or trauma. Management focuses on treating the underlying cause, restoring oxygen delivery, and stabilizing circulation with carefully selected intravenous therapies [5]. Type B lactic acidosis, however, is a metabolic disturbance characterized by elevated blood lactate levels unrelated to tissue hypoxia. It is categorized into three subtypes: B1, caused by specific diseases; B2, associated with toxins or drugs; and B3, linked to metabolic errors [9]. Treatment emphasizes supportive measures, including buffering agents, dialysis, and mechanical ventilation when needed, to preserve physiological function and promote lactate clearance. A summary of currently published case reports of various etiologies of type B acidosis in patients without malignancy is shown in Table 1.

Type B lactic acidosis secondary to malignancy was not included in Table 1 due to the extensive number of cases in the literature. However, malignancy is a very important cause of lactic acidosis that occurs via two mechanisms: anaerobic metabolism of tumor cells and the Warburg effect. The Warburg effect is a biochemical phenomenon wherein tumor cells preferentially shunt pyruvate towards lactate production, despite normal oxygen levels and functional mitochondria [32]. Tumor cell lactogenesis promotes cell survival and disease progression via several complex direct and indirect biochemical mechanisms [33]. The concerted loss of tumor suppressors, oncogene activation, and angiogenic upregulation, with altered metabolism and signaling within the tumor microenvironment, are early processes that establish the Warburg phenotype [33]. Although oxidative phosphorylation yields higher ATP per glucose level, the rate of ATP production per glucose level is higher in aerobic glycolysis than in oxidative phosphorylation [33]. As such, the Warburg effect is a net adaptive mechanism that allows malignant cells to produce and subsequently utilize sufficient energy to support high metabolic demands. Lactate is an oncometabolite byproduct of the Warburg effect that further amplifies proliferation and progression by extracellular acidification, which promotes angiogenesis, metastasis, and immunosuppression [34]. Lactic acidemia is a prognostic factor for disease burden and mortality, given the synergistic production of abundant energy molecules, and a carcinogenic byproduct from the Warburg effect sustaining and propelling carcinogenesis [35].

In our patient, the persistent elevation of lactate levels at presentation initially raised concerns for sepsis or shock, prompting close monitoring for any signs of clinical deterioration. However, throughout his hospital stay, the patient remained hemodynamically stable, alert, and non-hypoxic. Laboratory tests and chest X-ray findings were unremarkable, with past imaging revealing only a congenital portosystemic shunt in the liver. No evidence of septic or hypovolemic shock was identified, making type A lactic acidosis unlikely. An intriguing aspect of our patient’s case is his advanced age, as research suggests that aging may elevate the risk of lactic acidosis due to physiological declines in renal mass and creatinine clearance [36,37]. However, while this could be a contributing factor, the degree of lactic acidosis observed cannot be solely attributed to aging, given our patient appears to be well-compensated. A thorough review of the patient’s medical and surgical history ruled out other differential diagnoses, as summarized in Table 2. These findings collectively suggest that the patient most likely experienced type B lactic acidosis, attributed to a combination of increased lactate production from albuterol use and impaired lactate clearance due to the congenital portosystemic shunt.

Beta-agonists are a known cause of type B lactic acidosis, often in the setting of acute asthma treatment. A number of case reports related to beta-adrenergic uses have been described in the literature (Table 1). While the exact mechanism is unknown and likely complex, it is thought to involve stimulation of lipolysis leading to an increased concentration of free fatty acids that suppresses acetyl CoA production and shunts pyruvate towards lactic acid production [46]. Additionally, our patient received intravenous methylprednisolone and oral prednisone for asthma exacerbation, which may have amplified β-adrenergic receptor sensitivity due to the potentiating effects of steroids [47]. While asthma exacerbation and significant beta-agonist treatment may have factored into this patient’s elevated lactate levels, the persistent elevation of lactate following the resolution of his sepsis during the prior admission suggests an underlying clearance impairment due to the congenital portosystemic shunt.

Portosystemic shunts bypass normal hepatic blood flow, reducing the liver’s ability to eliminate metabolites such as ammonium and lactate [48]. In this patient, the congenital shunt likely impaired lactate clearance, explaining the sustained elevation in lactate despite the resolution of acute insults. While hepatic dysfunction at a cellular level, as seen in cirrhosis and Mauriac syndrome, is a well-documented cause of hyperlactatemia [44,49], it follows that structural hepatic abnormalities may also diminish lactate clearance. Despite the shunt, the patient did not exhibit symptoms of hepatic encephalopathy, such as altered mental status, indicating a small anatomical connection and compensated hepatic function. However, even a small shunt results in a constant diversion of portal blood flow away from the liver, bypassing the hepatic parenchyma where lactate metabolism primarily occurs. Over time, this diversion can have a cumulative effect, ultimately leading to a measurable impairment in lactate clearance. In the context of clinical stability, persistently elevated lactate suggests a chronic etiology, such as a congenital liver anomaly, rather than an acute process. To our knowledge, there are no prior reports of lactic acidosis attributable to structural liver malformations. This unique combination of increased lactate production (albuterol) with decreased clearance (congenital portosystemic shunt) creates the conditions for lactate elevation that is not entirely dependent on disease severity.

## 3. Conclusions

We present a unique case of an elevated lactate level in a patient with chronic beta-adrenergic agonist use and a congenital intrahepatic portosystemic shunt. Beta-adrenergic stimulation from his albuterol use likely led to increased lactate production, while his congenital portosystemic shunt may have impaired lactate clearance, leading to persistently elevated lactate. While beta-agonists are a recognized cause of type B lactic acidosis, a potential role for structural liver abnormalities in reduced lactate clearance must be examined further.

## Figures and Tables

**Table 1:** Summary of various causes of type B lactic acidosis.

Year	Etiologies	Age/Gender	Lowest pH	Highest Lactate (mmol/L)	Source	Treatment	Outcome
1984	Acetaminophen toxicity [10]	29/F; 48/F	6.98; 6.92	25.8; 17.6	Arterial; Serum	Bicarbonate, thiamine, dialysis	Death
1999	Zidovudine use [11]	34/F	6.77	26.8	Arterial	Supportive	Death
2000	Stavudine use [12]	35/F; 34/M; 55/F; 38/F; 50/F	7.3	10.3	Arterial	Bicarbonate, thiamine	Resolution
2001	TURP syndrome [13]	71/M	7.29	6.8	Arterial	Supportive	Resolution
2001	Nucleoside analog use [14]	51/F	7.35	5.03	Arterial	Riboflavin	Resolution
2002	Nucleoside analog reverse transcriptase inhibitor use [15]	47/F	7.1	265	Arterial	Bicarbonate, thiamine, riboflavin	Resolution
2008	Insulin resistant hyperglycemia [16]	14/M	7.31	10.3	Serum	Supportive	Resolution
2011	β-adrenergic agents [17]	49/F	7.29	10.47	Arterial	Discontinuation of beta-agonist	Resolution
2016	Venlafaxine overdose [18]	55/M	7.39	8.6	Arterial	IV fluids	Resolution
2017	Thiamine deficiency [19]	Neonate/F	NR	10.4	NR	Intravenous thiamine	Resolution
2017	Ombitasvir/ Paritaprevir/Ritonavir/Dasabuvir use [20]	64/F; 61/F; 59/M	NR	>15; >15; 5.9	NR	Hemofiltration; hemodialysis	Death; Resolution; Resolution
2018	Thiamine deficiency [21]	62/M	7.17	14.5	Arterial	Thiamine supplement	Resolution
2018	Albuterol use [22]	50/M	7.31	10.3	NR	Discontinuation of albuterol	Resolution
2019	Mauriac syndrome [23]	16/F	NR	13.43	NR	Glycemic control	Partial resolution
2019	End-stage liver disease [24]	16/F	NR	30.73	Arterial	Bicarbonate, hemofiltration	Death
2020	Albuterol use [25]	63/F	NR	6.7	NR	Discontinuation of albuterol	Resolution
2021	Thiamine deficiency [26]	63/F	7.15	24	Arterial	Thiamine supplement	Resolution
2021	Salbutamol use [27]	40/M	6.98	4.6	Serum	Intubation	Resolution
2022	Thiamine deficiency [28]	54/F; 42/M	6.94; 7.37	14; 20	NR; Arterial	Thiamine supplement	Resolution
2023	Caffeine intoxication [29]	23/M	7.3	6.26	NR	Supportive	Resolution
2024	Metformin use [30]	43/M	6.9	>30	Arterial	Hemodialysis and tris-hydroxymethyl aminomethane	Resolution
2024	Cannabinoid use [31]	42/M	7.18	25.6	Arterial	Hemodialysis	Resolution

NR: not reported; NA: not applicable.

**Table 2:** Etiologies of type B lactic acidosis and exclusion rationale.

Differential Diagnosis	Mechanism	Reason for Exclusion	Ref.
Short-bowel syndrome and other malabsorptive conditions	Buildup of undigested carbohydrate fosters growth of D-lactate-producing bacteria	No surgical history or clinical evidence of malabsorption	[8]
Malignancy	Multiple mechanisms including anaerobic metabolism by tumor cells and the Warburg effect	No fatigue, weight loss, cytopenia, or incidental findings on imaging. No evidence of cancers in labs and imaging studies	[32]
Inborn errors of metabolism	Alterations in lactate production and utilization	No childhood symptoms	[38]
Seizures, shivering, intense exercise, acute asthma	Hypermetabolic states	No history of seizures, shivering, or intense exercise	[39,40]
Diabetes	Biguanides like metformin inhibit hepatocyte mitochondrial respiration leading to decreased lactate clearance. Patients in diabetic ketoacidosis may have lactic acidosis independent of biguanides.	No history of diabetes	[41,42]
Thiamine deficiency	Shunting of pyruvate to anaerobic metabolism due to lack of pyruvate dehydrogenase complex cofactor	No history of alcoholism. Normal thiamine level. No signs of Wernicke’s encephalopathy or Korsakoff’s syndrome	[43]
Cirrhosis	Impaired lactate clearance secondary to liver disease	No constitutional symptoms or physical exam findings. Normal liver function	[44]
Antiretroviral therapy (ART)	Drug-induced mitochondrial toxicity	No history of ART use	[45]

## Data Availability

The datasets used and/or analyzed during the current study are available from the corresponding author upon reasonable request.
